# Synovial fibroblast-miR-214-3p-derived exosomes inhibit inflammation and degeneration of cartilage tissues of osteoarthritis rats

**DOI:** 10.1007/s11010-022-04535-9

**Published:** 2022-08-24

**Authors:** Chenteng Lai, Boyi Liao, Song Peng, Peng Fang, Nirong Bao, Lei Zhang

**Affiliations:** 1 Department of Orthopedics, Jinling Hospital, Nanjing University, School of Medicine, No. 305 East Zhongshan Road, Nanjing, 210002 China; 2 Department of Orthopedics, The People’s Hospital of Wugang City, Wugang, 422400 China

**Keywords:** miR-214-3p, Exosomes, Osteoarthritis, Inflammation, Cartilage tissue

## Abstract

MicroRNAs (miRs) are regulators of number of cellular process. miRs enclosed within exosomes can be crucial regulators of intercellular signalling and could be an important biomarker of various age-associated disorders. Role of exosomal enclosed miRs in osteoarthritis (OA) chondrocytes and synovial fibroblasts (SFBs) remains poorly studied. Here, we profiled and studied the effect of synovial fluid-derived exosomal miRs on inflammation, survival, proliferation of chondrocyte in correlation with cartilage degeneration. Exosomes were isolated from synovial fluid collected from OA subjects and were analysed by transmission electron microscopy. miRs were isolated and were submitted to microarray profiling. Web-based PCR analysis was done. Chondrocyte proliferation and colony formation assay were performed. Apoptosis study was done by flow cytometer. Gene expression was done by qRT-PCR analysis and protein expression by western blot assay. Rat model of OA was created by operating the knee by anterior cruciate ligament and resection of medial menisci (ACLT + MMx) method. Micro-CT analysis, histological analysis, immunohistochemical staining, and TUNEL assay were also performed. About 17 miRs were found to be expressed differentially in the synovial fluid collected from the control and OA subjects. Microarray analysis confirmed, expression of miR-214-3p was significantly downregulated in the synovial fluid exosome of OA subjects. miR-214-3p mimic promoted proliferation of chondrocyte and suppressed apoptosis. Treatment also inhibited the levels of TNF-α, IL-1β and IL-6. SFB-miR-214-3p exosomes suppressed apoptosis and also inflammation in chondrocytes. In vivo study suggested that SFB-exosomal miR-214-3p from rats suppressed the formation of osteophytes, prevented degeneration of cartilage and exerted anti-inflammatory and anti-apoptotic effect in articular cartilage tissue. The findings suggested that SFB-miR-214-3p exosomes can ameliorate chondrocyte inflammation and degeneration of cartilage tissues. The study confirms therapeutic potential of SFB-miR-214-3p exosomes in treating OA.

## Introduction

Osteoarthritis (OA) is an inflammatory disorder characterised by cartilage degradation, synovial inflammation and remodelling of subchondral bone leading to poor life quality [1, 2]. Acute synovitis is the initial stage change associated with increased levels of pro-inflammatory mediators observed in subjects with OA [3, 4]. Hence to study and monitor the pathophysiological alterations responsible for cartilage and joint-associated problems, samples of synovial fluid are analysed. SFBs are responsible for secreting synovial fluid which is responsible for lubricating the articular cartilage [5, 6]. OA is characterized by increased expression of pro-inflammatory factors and cytokines such as tumour necrosis factor (TNF), interleukin-1β (IL-1β), IL-2 and IL-7 [7–9]. These mediators are responsible for differentiation and viability of chondrocytes, also they are responsible for activation of matrix metalloproteinases (MMP) and aggrecans [9]. Use of anti-inflammatory therapies in treating the inflamed synovial joint condition is considered a favourable approach in preventing the progression of OA.

Exosomes are extracellular nano-vesicles present in the circulatory system responsible for cellular communication process and also responsible for regulating various biological processes. Exosomes are carriers and miRs are one of the cargos which is transported by them [10]. In addition to miRs, exosomes carry various mRNAs which are transported to the cells by fusion process of cell membrane. Exosomes have recently been identified to be important mediators responsible for interaction between the cells [11]. The proteins and miRs carried by exosomes have been reported to affect the differentiation and survival of cells which affect the progression and development of OA [12, 13]. The exosomes are released by the myriad cells and tissues and SFB. Synovial fluid of osteoarthritis patients has been detected to show the presence of neutrophils-derived exosomes [14]. These exosomes influence the pro-inflammatory pathways which govern degradation of extracellular matrix and angiogenesis [15, 16]. Exosomes present in SFBs are the important regulators of local inflammation of joints mediated by SFBs and chondrocytes due to their property to transport miRs and inflammatory proteins to tissues [17].

Recent reports suggest therapies for OA targeting miRs which are involved in regulating the chondrocyte functions [18–20]. In synovial fluid, miRs are prone to rapid degradation; however, exosomes prevent this degradation by stabilizing them and hence improving their property of induced differentiation, survival and proliferation in cells [21]. In a report earlier, synovial fluid miR-210 has been found to be an important biomarker of OA [22]. miR-29b-3p was found to overexpressed in synovial fluid of OA patients [23]. Also, in a study earlier, miR-200-c was found to overexpressed in synovial fluid-derived exosomes of OA patients [24]. Such exosomes which have pro-chondrogenic miRs within them could be vital substitute for damaged chondrocytes in osteoarthritis [25]. Research directed towards finding some specific miRs which would control the process of chondrogenesis can be helpful in developing therapies for countering OA.

Synovial fluid is viscous fluid present in the joints for reducing the friction between them. The fluid is rich in exosomes and some important inflammatory proteins hence it can serve as a very important determiner of the exact pathology behind the development and progression of OA [26]. By screening the miRs present in the exosomes of synovial fluid, we can get a better understanding about the signalling pathways and the mechanism involved behind the progression of OA, also it would help in identifying the potential targets for treatment.

MiR-214-3p is miRNA widely distributed in vertebrates, and it is found to be associated with hepatic gluconeogenesis [27]. The miR-214-3p has also been associated with skeletal disorders, by suppressing osteogenic differentiation of myoblast cells via targeting the osteoblast-specific transcription factor Osterix [28]. A report also found that miR-214-3p targets ATF4 which is an important osteogenic transcriptional factor involved in suppressing bone formation [29]. Also, in a study, miR-214-3p was found to promote osteoclastogenesis via PI3K/Akt pathway involving the PTEN protein [30]. It was reported that miR-214-3p was suppressed in IL-1β-induced OA mice model, the study confirmed potential involvement of miR-214-3p in OA in vivo [31]. However, the role of exosomal miR-214-3p in OA remains unexplored. Here, we evaluated the functional relevance of exosomal miR-214-3p for understanding its involvement as suppressor of SFBs induced inflammation of chondrocyte and degradation of cartilage.

## Materials and methods

### Sampling

**Fig. 1:**
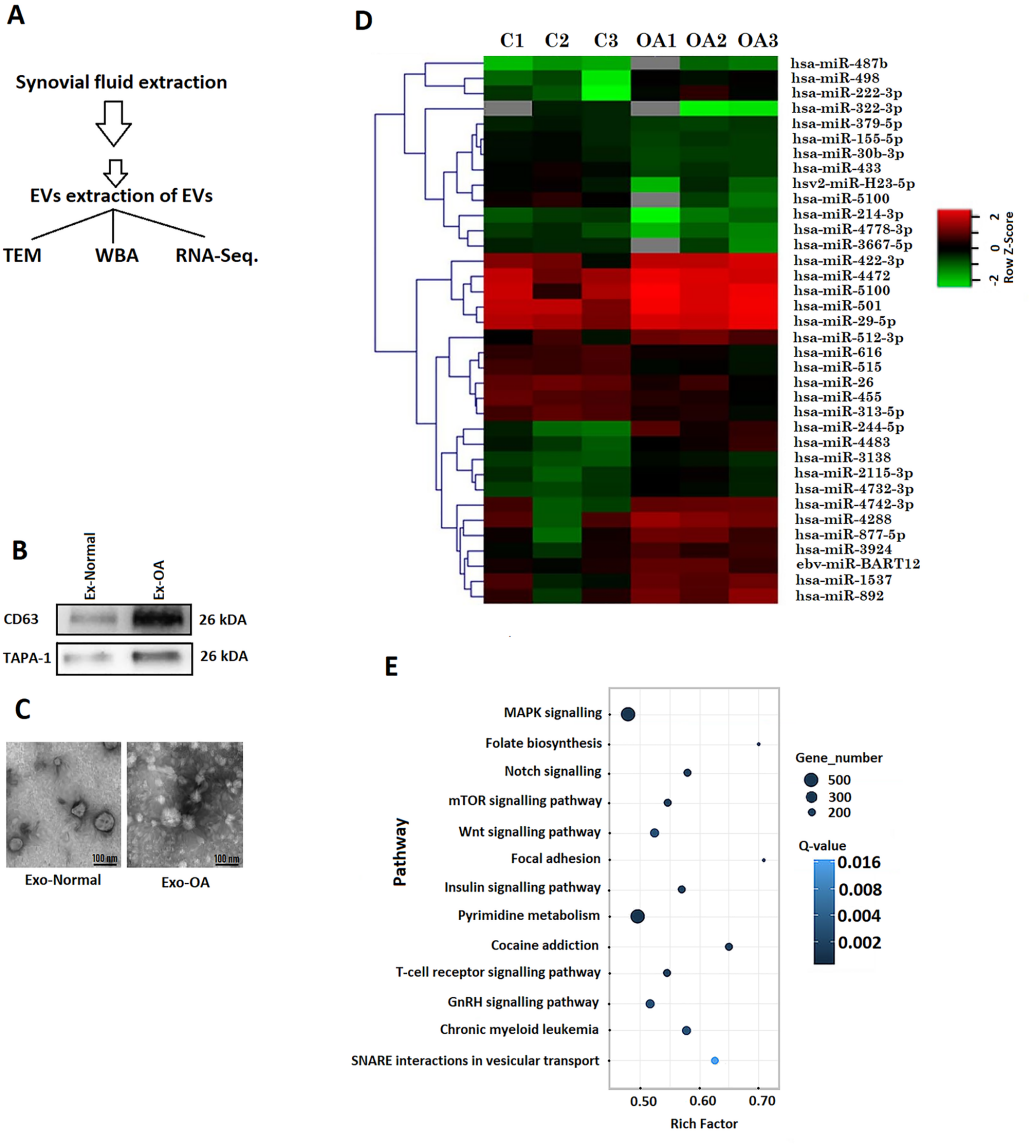
Bioinformatic analysis of exosome encapsulated miRs. **A** Procedure for collecting synovial fluid from knee of human volunteers. **B** Western blot analysis of CD63 and TAPA-1 in exosomes like vesicles. **C** Transmission electron microscopic morphological analysis of exosomes like vesicles. **D** Heatmap showing expression of miRs in synovial fluid exosomes of OA subjects. **E** Annotations of KEGG pathway analysis showing various pathways

About 12 patients (6 females, 6 males) suffering from OA and enrolled for total knee replacement ageing between 60 and 65 years were included in the study, and the synovial fluid samples were collected from the patents. Similarly normal subjects (*n* = 12) ageing between 60 and 65 years with no complaint for OA were also included, and synovial fluid sample was collected from them also. Prior to study, the patients and normal subjects were informed about the study and written consent was obtained from them, and the study was approved by the human ethical review board of Jinling Hospital, Nanjing University, School of Medicine, China (approval no. NU04CE). The synovial fluid samples collected were immediately processed in laboratory for isolating exosomes (Fig. 1A).

### Isolation of exosome-like vesicle

The exosomes were isolated by ultracentrifugation, and briefly, the synovial fluid samples (Fig. 1A) and supernatants of SFB were centrifuged in a cooling centrifuge (4 °C) at 300×*g* for 10 min, and the pellet formed at the bottom was discarded. The supernatant was again centrifuged at 2000×*g* for 10 min followed by centrifugation at 10,000×*g* for 30 min, again the supernatants were centrifuged for 100,000×*g* for 60 min, and the exosomes were obtained as pellet at bottom, which was suspended in PBS and the suspension was analysed using nanoparticle tracking analysis (NanoSight NTA systems, Malvern) and transmission electron microscopy Talos L120C TEM (ThermoFisher USA).

### Isolation of exosomal miRNA and microarray study

For isolating the miRNAs from obtained exosomes, Ambion PureLink miRNA Isolation Kit (ThermoFisher USA, Catalog# K157001) was used as per supplied instructions. Quantity and quality of isolated miRs were done by using Spectrophotometer (ThermoFisher USA) and a Bioanalyzer (Agilent, USA) at absorbance maxima of 260 nm. The profiling of exosomes-derived miRs was done using GeneChip miRNA 4.0 Array (Applied biosystems, ThermoFisher USA, Catalog# 902411). The heat maps for miRs were generated using the hierarchical clustering method.

### RT-PCR analysis of differential expressed miRs

A web-based PCR array analysis was done using the GeneGlobe Data Analysis Centre (Qiagen USA). The miR PCR data of control and OA groups were normalized as per the procedure described earlier [32]. The data outputs were analysed for differentially expressed miRs from both normal and OA subjects statistically by *t* test, and the fold changes of miRs greater than 1.5 were included in the study. The miRs of both normal and OA group with Ct values > 35 were rejected and were considered undetected from study so as to minimise the noise of measurements. The differentially expressed miRs were then submitted for DIANA tools using miRpath v.3 as web-based application (http://snf-515788.vm.okeanos.grnet.gr/). The results were further submitted to principal component analysis.

### Cell culture and transfection

The Sprague–Dawley rats (SD) were used; the knee joints were isolated and were submitted to enzymatic digestion for isolating primary SFB and chondrocytes. The rats were supplied by the animal centre of Jinling Hospital, Nanjing University, School of Medicine. The synovial tissue and cartilage tissues were treated with 0.25% trypsin for 1 h and EDTA 0.02% and then divided into small pieces of 0.5 to 1 mm^3^, and the pieces were then treated with collagenase-II (0.2%) for 4 h at room temperature conditions. The cells were collected and suspended in DMEM media containing fetal bovine serum (10%), and the cells were then cultured at room temperature with 5 CO_2_. For transfecting, the cells were suspended in DMEM/F12 media added with fetal bovine serum and plated in 96-well plates and incubated under humid conditions with 5% CO_2_ for 24 h until the cells reached confluence of 70%, after this the cells were transfected with miR-214-3p mimic or inhibitor (100 pM) as per supplied instructions. The animal study protocols were approved by the animal ethical committee of Jinling Hospital, Nanjing University, School of Medicine.

### Colony formation and proliferation studies

Chondrocyte colony formation study was done to evaluate the role of miR-214-3p mimic or inhibitor on proliferation of isolated chondrocytes. Briefly, about 5 × 10^2^ cells were transferred to culture plates for 24 h and were cultured for 2 weeks in DMEM media; after 2 weeks, the cells were rinsed with PBS and fixed for 20 min in formalin (10%) followed by crystal violet staining (0.5%) for 1 h. The images were recorded with digital microscope for counting the total colony numbers.

For chondrocyte proliferation, CCK-8 assay was performed to study the effect of miR-214-3p or SFB-miR-214-3p exosomes on proliferation of chondrocytes. The isolated rat chondrocytes (0.5 × 10^4^ cells/well) were transferred to 96-well plates and treated with sodium nitroprusside (0.75 mM) followed by the treatment of various concentrations of miR-214-3p or SFB-miR-214-3p exosomes for 24 to 48 h and proliferation was evaluated. The wells were added with CCK-8 reagent 100 μl (10%) for 2 h, the wells were studied by microplate reader, and the optical activity was observed at 450 nm.

### Apoptosis analysis

For apoptosis study, we performed Annexin V-FITC analysis (ThermoFisher USA, Catalog# 331200) as per supplied instructions. Briefly, the chondrocytes were centrifuged at 1000×*g* for 5 min two times in cold PBS and then suspended in binding buffer (500 μl) having 5 μl each of Annexin V-FITC and PI. The chondrocytes were then incubated for 15 min in dark and were then submitted for flow cytometry analysis (FACScan Beckton Dickson USA), and apoptosis rate was studied with flow cytometer.

### Quantitative real-time polymerase chain reaction (qRT-PCR)

The total RNA was isolated using Trizol reagent (ThermoFisher USA, Catalog# 15596026) from miRs or chondrocytes-treated exosomes as per the provided instructions. The RNAs were characterized quantitatively and qualitatively using a spectrophotometer and then cDNA was prepared using PrimeScript RT kit. For qRT-PCR analysis, SYBR premix (Takra Biotech USA, Catlog# RR420A) was used along with qTOWER qRT-PCR system (AnalytikJena USA). GAPDH was selected as loading control, whereas the analysis was carried by 2^−ΔΔCT^ method for studying the expression of gene.

### Western blot analysis

The extracellular vesicles were submitted to lysis using the RIPA buffer added with protease inhibitor cocktail. The resultant was analysed for total proteins using the protein estimation kit (Sigma Aldrich USA, Catlog# 51254). The proteins were incubated with specific antibodies overnight for obtaining blots. The antibodies used were specific for IL-6 (1:500) (ab6672), IL-1*β* (1:500) (ab9722), TNF-α (1:500) (ab6671), CD81 (1:1000) (ab109201), CD63 (1:1000) (ab231975), CD9 (1:1000) (ab223052) and HSP70 (1:1000) (ab2787), all the antibodies were obtained from Abcam USA. The blots were detected using Western blot detection system (Thermo Fisher USA).

### Animal studies

For the study, Sprague Dawley rats (male) weighing between 220 and 250 g were obtained from the animal centre of Jinling Hospital, Nanjing University, School of Medicine, China. The animal experiments were approved by the Jinling Hospital, Nanjing University, School of Medicine, China (Approval Number: NU0031AE). The animal studies were done to study the effect of SFB miR-214-3p exosomes on the cartilage tissues of rats. The animal model of OA was created by the ACLT+ MMx as described earlier with minor modifications [33]. Briefly, the rats were submitted to pentobarbital anaesthesia (20 mg/kg), the right knee was operated for anterior cruciate ligament and resection of the medical menisci, and the sham rats did not undergo resection. The rats were divided into Sham operated, OA control, SFB-miR-214-3p exosomes, OA+ SFB-control exosome group and OA+ SFB-miR-214-3p exosomal group. After 1 week of operating the rats, they were shifted to rotating cage for 15/day as described earlier [34]. After 4 weeks post-surgery, the rats were injected (intra-articular route) with defined treatments according to groups once in a week, and the sham-operated group was injected with PBS. After 10 weeks of study, the rats were submitted to micro-CT, TUNEL assay and histopathology.

### In vivo micro-CT analysis

In vivo micro-CT analysis was performed using micro-CT systems Bruker Germany for analysing the knee joints. The images were gathered and evaluated for the structure of joints and quantified for mean trabecular thickness (Tb. Th, mm), morphometric indices by measuring the bone volume fraction i.e. the ratio of bone volume/total volume (BV/TV, %) and mean trabecular separation (Tb. Sp, mm).

### Histological studies

The rat knee joints were disarticulated and the femoral condyle and tibial plateau were fixed in para formaldehyde (4%) for 24 h. The tissues samples were decalcified by treating them with EDTA (10%) pH 7.4 for 21 days, and then the tissue samples were embedded in paraffin. The tissues samples were then processed in rotary microtome to obtain tissues sections of 5 mm and submitted to haematoxylin and eosin staining (H&E).

### TUNEL assay

The cell apoptosis was done by TUNEL assay with the help of cell death detection kit (ThermoFisher USA) (Catlog# V13242) as the supplied instructions. Briefly, the cartilage tissue isolated from knee joint was exposed to proteinase solution (20 µg/ml) for 15 min, after then, the apoptotic chondrocytes were processed for labelling. The apoptotic cells were quantified using a microscope.

### Immunohistochemical analysis

The expression levels of TNF-α and IL-1β in cartilage tissues were analysed by immunohistochemical analysis. Briefly, the cartilage tissue sections were incubated for 12 h at 4 °C with primary antibodies. The sections were submitted to diaminobenzidine staining kit (Sigma Aldrich USA) (Catlog#D7304) for detecting the binding of antibodies, and then, the sections were counterstained with haematoxylin staining. The sections for staining were evaluated by optical microscope, and the images of cells were recorded and processed with Image-Pro software.

### Statistical analysis

The results were presented as mean ± % relative standard deviation (RSD), and the comparison was done via one-way ANOVA, post hoc analysis was done by student *t* test. All the statistics was performed using GraphPad prism Software. Value *P* < 0.05 was confirmed to be significant.

## Results

### Human synovial fluid exosomes

The synovial fluid samples collected from OA and normal subjects were processed for isolating exosomes using the exosome isolation reagent. The exosomes were submitted to immunoblotting analysis and were confirmed to have significant expression of TAPA-1 and CD63 (Fig. 1B). The exosomes showed round structure when observed under TEM (Fig. 1C).

### Expression of miRs in synovial exosomes

The synovial exosomes obtained from OA and normal subjects were analysed for miRs, and it was found that among the total 17 miRs, 7 were downregulated (miR-214-3p, miR-4778-3p, miR-3667-5p, miR-422-3p, miR-4472, miR-5100 and miR-29-5p) and 10 were upregulated (miR-498, miR-222-3p, miR-379-5p, miR-155-5p, miR-30b-3p, miR-433, miR-512-3p, miR-616, miR-515 and miR-26) in OA patients compared to normal subjects. Supervised clustering approach was utilized for creating heat map which showed variations in the samples of normal and OA subjects. The results clearly suggested variable expression of about 17 miRs in the synovial exosomes of OA subjects compared to normal (Fig. 1D). It was found that of the differentially expressed miRs, the expression of miR-214-3p was decreased significantly in the synovial exosomes in OA (OA-exosomes) subjects compared to exosomes from normal subjects (non-OA-exosomes).

### KEGG pathway analyses of variably expressed miRs

For evaluating the functional role of differentially expressed miRs, we compared OA-Exosomes and Non-OA-Exosomes with the help of KEGG functional enrichment analysis. The results of KEGG pathway analysis suggested that the variably expressed miRs were linked to various pathways associated with signal transduction, cell migration, cell proliferation and metabolism (Fig. 1E). The KEGG pathway analysis identified pathways such as MAPK signalling, Folate biosynthesis, Notch signalling, mTOR signalling pathway, Wnt signalling pathway, Focal adhesion and insulin signalling pathway (Fig. 1E). The KEGG analysis suggested involvement of miR-214-3p with some specific OA-specific pathways such as MAPK, Wnt and mTOR.

### miR-214-3p encourages proliferation of chondrocytes and inhibits inflammation and apoptosis

**Fig. 2:**
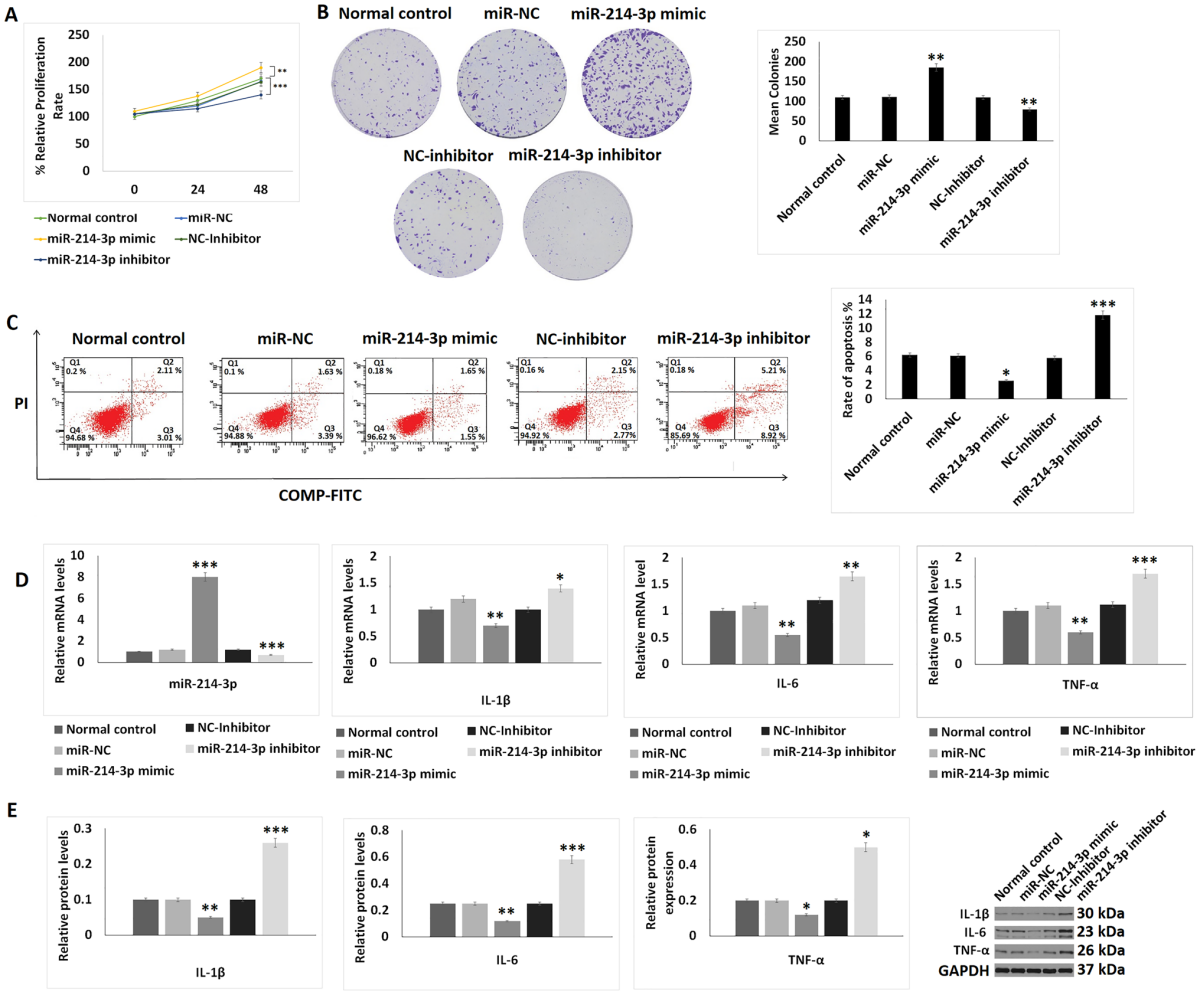
Effect of miR-214-3p on proliferation, apoptosis and inflammatory response on chondrocytes. **A** CCK-8 analysis showing effect of miR-214-3p mimic and inhibitor on cell proliferation for 24 and 48 h. **B** Colony formation assay on chondrocytes exposed to miR-214-3p-mimic or inhibitor. **C** Flow cytometry analysis for studying apoptosis index after exposure to miR-214-3p mimic or inhibitor for 24 h. **D** qRT-PCR analysis for relative mRNA levels of miR-214-3p, IL-1β, IL-6 and TNF-α after exposure to miR-214-3p mimic or inhibitor for 24 h. **E** Western blot analysis for expression of inflammatory proteins IL-1β, IL-6 and TNF-α after exposing the chondrocytes to miR-214-3p mimic or inhibitor. The results are presented as mean (*n* = 3) ± %RSD. *P < 0.05, **P < 0.01, ***P < 0.001 compared to normal control. Groups normal control, NC-inhibitor (negative control-inhibitor), miR-NC (miR-negative control), miR-214-3p mimic, miR-214-3p-inhibitor

As it was found that the expression of miR-214-3p was downregulated significantly in OA patients-derived exosomes, we postulated that miR-214-3p may be associated with the pathogenesis of OA. The cell proliferation studies by CCK-8 assay suggested that the miR-214-3p mimics transfected chondrocytes showed improved proliferation compared to control chondrocytes; however, the miR-214-3p inhibitor transfected group showed opposite results i.e. decreased proliferation of chondrocytes (Fig. 2A). Then, we studied the effect of miR-214-3p on the proliferation of chondrocytes by performing the colony formation assay. The outcomes suggested that, transfection of miR-214-3p mimic in chondrocytes increased the colony formation compared to control cells, whereas the chondrocytes transfected with miR-214-3p inhibitor showed decrease in extent of colony formation (Fig. 2B). The results of Annexin V FITC/Propidium Iodide staining on apoptosis of chondrocytes suggested that, upon transfection of miR-214-3p mimic decreased the rate of apoptosis and the miR-214-3p inhibitor increased apoptosis compared to control (Fig. 2C). It was also found that the mRNA as well as protein levels of TNF-α, IL-1β and IL-6 were decreased significantly in chondrocytes transfected with miR-214-3p mimic and increased after miR-214-3p inhibitor transfection (Fig. 2D, E).

### SFB-miR-214-3p exosomes inhibited inflammation and apoptosis in OA chondrocyte

**Fig. 3:**
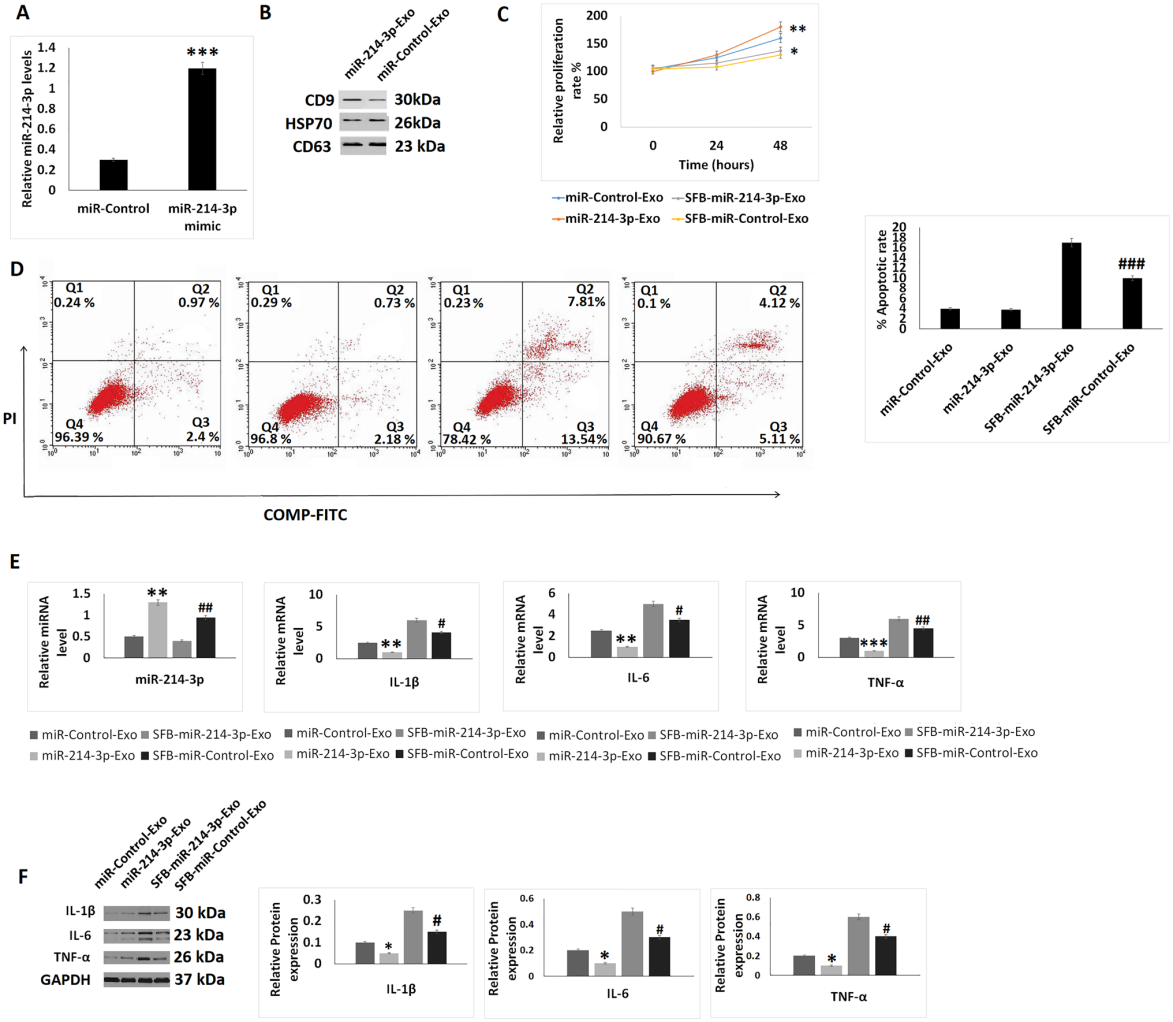
Effect of SFB-derived exosomes like vesicles on inflammation and apoptosis in chondrocytes. **A** qRT-PCR analysis for relative mRNA expression levels of miR-214-3p in SFB transfected with miR-214-3p mimic after 24 h of incubation. **B** Relative protein levels by western blot analysis for expression of CD9, HSP70 and CD63 in exosomes like vesicles. **C** CCK-8 analysis for cell proliferation after treatment with SFB-miR-214-3p-exo for 24 and 48 h. **D** Apoptosis study using flow cytometer after treatment of SFb-miR-214-3p-exo for 2 h before treatment of SNP for 24 h. **E** qRT-PCR analysis for relative mRNA levels of miR-214-3p, IL-1β, IL-6 and TNF-α after treatment of SFB-miR-214-3p-exo for 2 h prior to SNP treatment of 24 h. **F** Western blot analysis for protein levels for expression of inflammatory proteins IL-1β, IL-6 and TNF-α after treatment of SFB-miR-214-3p-exo for 2 h prior to SNP treatment of 24 h. The results are presented as mean (*n* = 3) ± %RSD.*P < 0.05, **P < 0.01 and ***P < 0.001 compared to miR-control-exo (miR-control-exosomes), ^#^*P *< 0.05, ^##^*P *< 0.01, ^###^*P* < 0.001 compared to SNP+ miR-control-exo

Morphological analysis of SFB-miR-214-3p exosomes was done and it was found that the exosomes were having approximate diameter of 100 ± 5 nm which was in agreement to the features of exosomes. The miR-214-3p mimic transfected the SFB which was evidenced by high expression levels of miR-214-3p in them (Fig. 3A). HSP70, CD9 and CD63 are the important protein markers expressed by exosomes, and our results showed that these markers were expressed by the SFB-miR-214-3p exosomes (Fig. 3B). The CCK-8 assay suggested that the SFB-miR-214-3p exosomes-treated chondrocytes showed increased proliferation upon treatment with or without sodium nitroprusside compared to control chondrocytes (Fig. 3C). The apoptosis rate in sodium nitroprusside-treated SFB-miR-214-3p exosomes was significantly lower compared to sodium nitroprusside and control SFB-exosomes, not showing the expression of miR-214-3p (OA+ SFB-control exosome) (Fig. 3D). On evaluating the effect of treatments on levels of TNF-α, IL-1β and IL-6, it was observed that treatment of SFB-miR-214-3p exosomes resulted in overexpression of miR-214-3p and inhibition of TNF-α, IL-1β and IL-6 at both protein as well as mRNA levels (Fig. 3E, F) compared to SFB-control exosomes-treated chondrocytes.

### SFB-miR-214-3p exosomes maintained the subchondral bone structure in OA rats

**Fig. 4:**
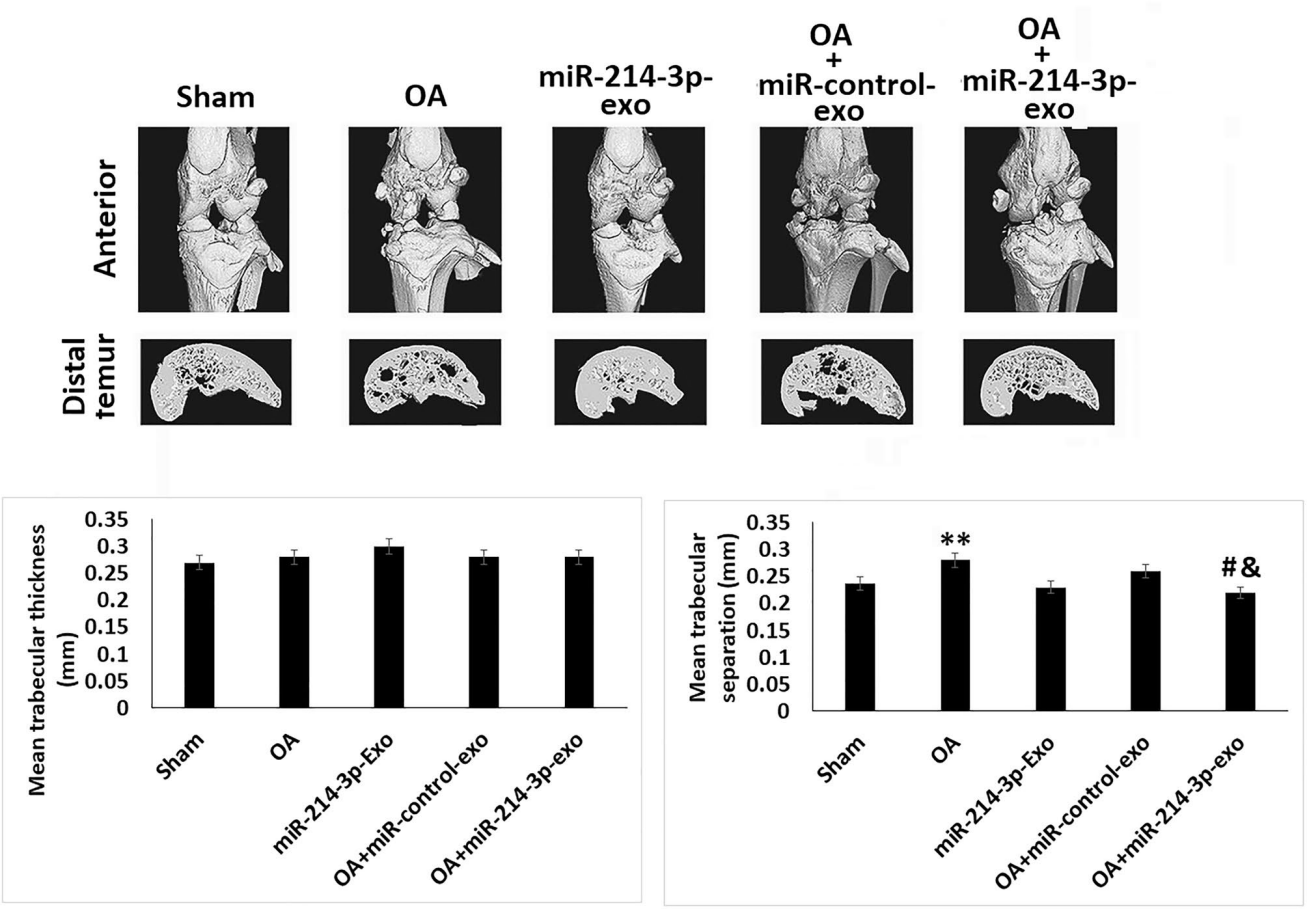
SFB-miR-214-3p exosomes maintained the subchondral bone structure in OA rats. Micro-CT analysis showing bones of rats subjected to ACLT+ MMX OA. Quantitative analysis showing mean trabecular thickness and mean trabecular separation. The results are presented as mean (*n* = 3) ± %RSD. ^*^P < 0.05 and **P < 0.01 compared to sham group, ^#^*P* < 0.05 compared to OA rats, ^&^*P* < 0.05 compared to OA + miR-control-exo rats

For studying the effect of SFB-miR-214-3p on bone structure, micro-CT analysis was done. For the in vivo study, a rat model of OA was developed, the model was used to study any alterations in cartilages, subchondral bone such as joint space, changes in calcification and formation of osteophyte (Fig. 4). It was found that the OA rats showed significantly decreased BV/TV values and increased Tb. Sp compared to sham-operated rats. Interestingly, the treatment of SFB-miR-214-3p exosomes reversed these changes, thus confirming that SFB-miR-214-3p exosomes maintained the structure of subchondral bone of OA rats.

### SFB-miR-214-3p exosomes inhibited the synovial inflammation induced degeneration of cartilage and synovium in OA rats

**Fig. 5:**
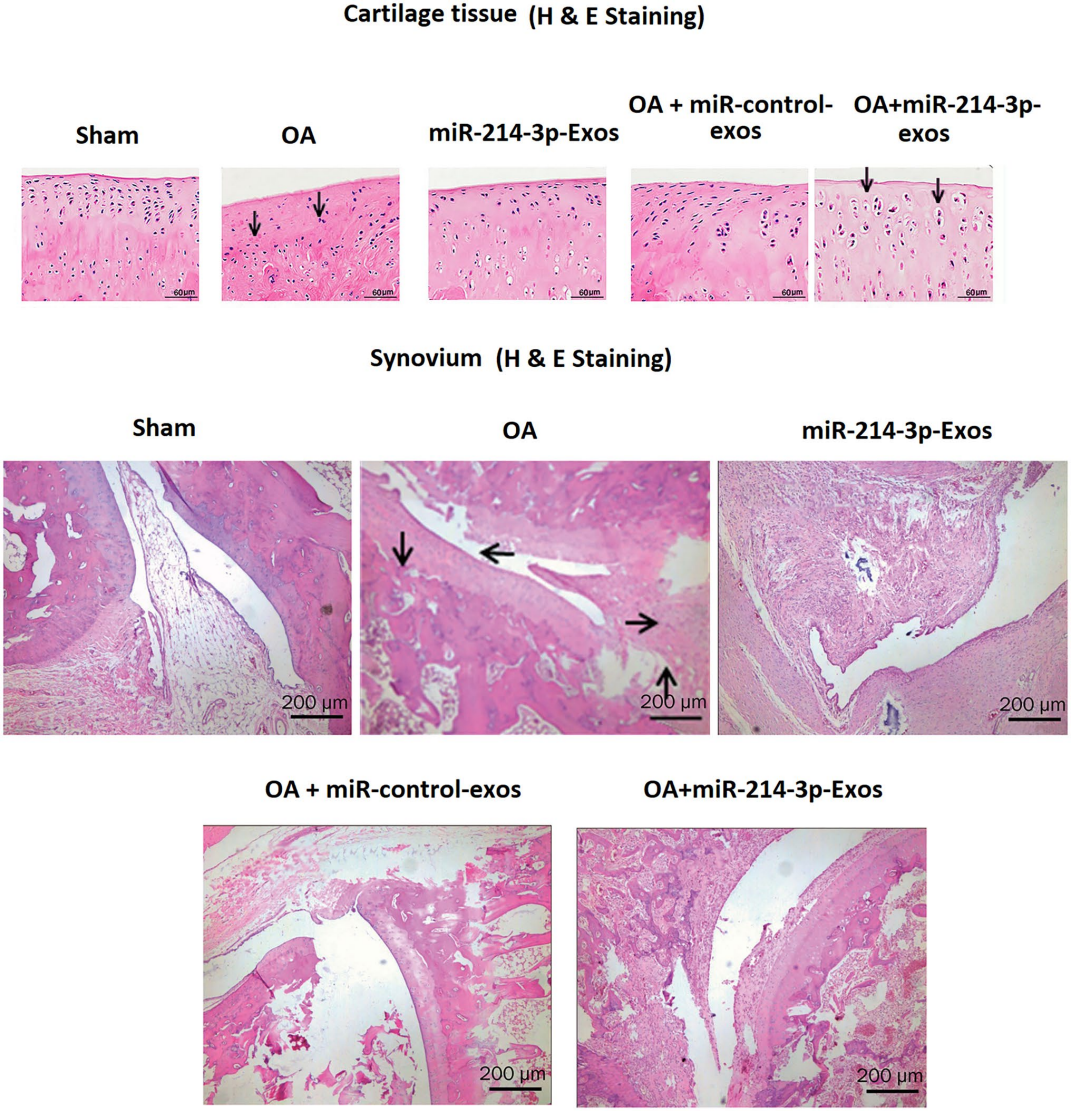
SFB-miR-214-3p exosomes inhibited the synovial inflammation-induced degeneration of cartilage and synovium in OA rats. H&E staining of cartilage or synovium of OA rats treated with SFB-miR-214-3p-exo showing improved cartilage thickness and regularity

The histological study in OA rats was done by H&E staining (Fig. 5). It was observed that against the sham-operated rats, the OA rats showed irregular articular morphology of cartilages, whereas OA rats treated with SFB-miR-214-3p exosomes showed enhanced thickness of cartilages and surface regularity which was associated with less severe cartilage degradation. The OA rats showed synovial thickening which was significantly decreased in OA+ SFB-miR-214-3p exosomes-treated rats, and the findings were in agreement with the property of SFB-miR-214-3p exosomes to decrease the inflammation of synovial joints.

### SFB-miR-214-3p exosomes suppressed the apoptosis and inflammation of chondrocytes of articular cartilage in OA rats

**Fig. 6:**
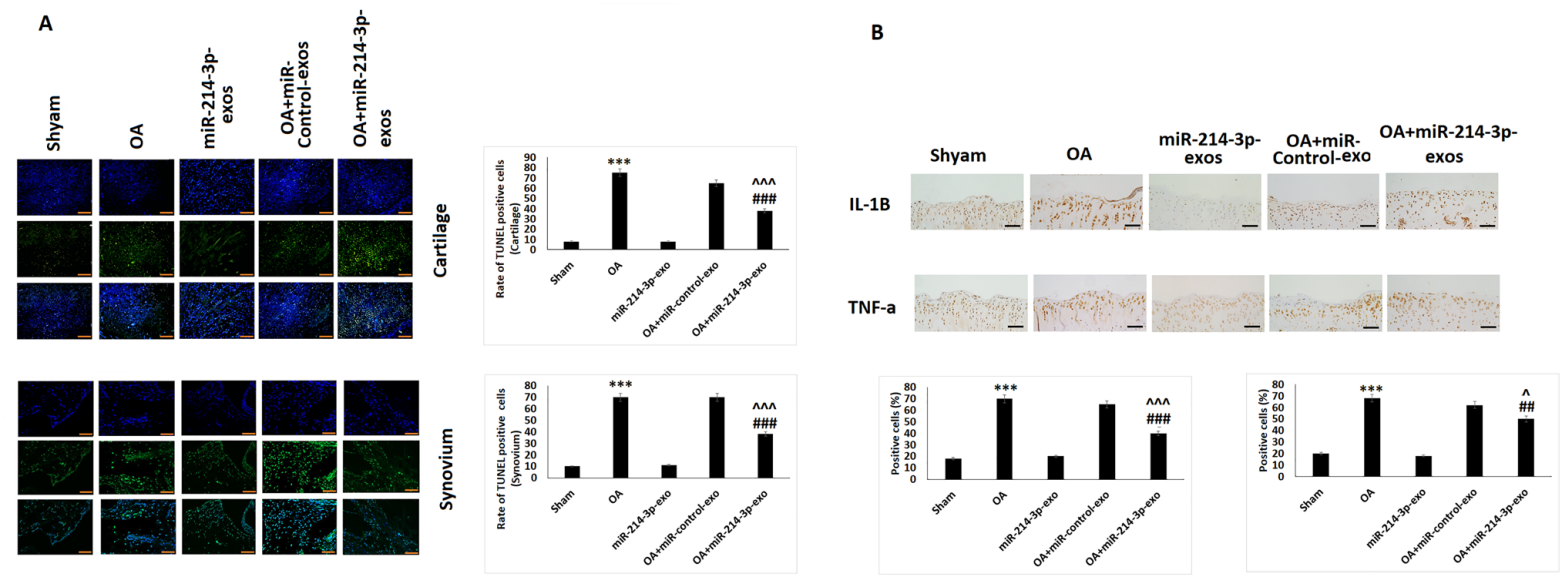
SFB-miR-214-3p exosomes inhibited apoptosis and inflammation in chondrocytes of articular cartilage in OA rats. **A** TUNEL analysis of cartilage and synovium for assessing apoptosis. **B** Immunohistochemical analysis for analysis of IL-1β and TNF-α in cartilage and synovium of OA rats. The results are mean ± %RSD. ***P < 0.001compared to sham rats, ^##^*P* < 0.01, ^###^*P *< 0.001 compared to OA rats, ^^^*P* < 0.05, ^^^^^*P* < 0.001 compared to OA + miR-control-exo group

The effect of SFB-miR-214-3p exosomes treatment on apoptosis of chondrocytes and SFB in rat model of OA was evaluated by TUNEL assay. Compared to sham rats, the samples of OA-induced rats showed significantly high number of TUNEL positive cells in cartilage (Fig. 6A) as well as in the synovial tissues (Fig. 6A). However, the chondrocytes treated with SFB-miR-214-3p exosomes showed decreased number of TUNEL positive cells. The immunohistochemical staining analysis was done to evaluate the expression of TNF-α and IL-1β in cartilage tissue sections of OA rats. The findings suggested that the expression of TNF-α and IL-1β was significantly decreased in SFB-miR-214-3p exosomes-treated OA rats compared to OA control rats (Fig. 6B).

## Discussion

The present work evaluated the potential of synovial fluid-derived exosomal miR in modulating the pathogenesis of OA and its effect on degeneration of knee joints of OA rats. We performed miR microarray analysis and found many miRs were expressed differentially in synovial exosomes form OA subjects. It was found that from the 17 variably expressed miRs, the major miRs downregulated were miR-432-5p, miR-138, miR-140, miR-204, miR-211 and miR-214-3p, whereas the miRs upregulated were miR-146a, miR-132, miR-180-a, miR-181-c and miR-181-d. Among all exosomal miRs, miR-214-3p was one of the most downregulated (about threefold) in the synovial fluid samples of OA subjects compared to the normal subjects, also prior reports had suggested involvement of miR-214-3p in OA condition [31].

The synovial fluid which is in close contact with articular cartilage and the synovial membrane present in the knee joints can serve as an important indicator of any pathological condition [35, 36]. Cell-derived exosomes are important indicators for studying the progression of any disease [37, 38]. Hence, in the present work, we performed analysis of miRs present within the exosomes isolated from the synovial fluid of osteoarthritis and normal subjects. Morphological analysis confirmed these particles to be exosomes looking into their diameters, also it was found that these particles were present at same levels in the synovial fluid in these subjects. It was also found that the exosomes had significant levels of miRs in them.

We next performed KEGG pathway analysis for target genes to screen the functional characteristics of miRs which were variably expressed in OA subjects. This screening suggested that the exosomal miRs were associated with some important pathways mainly associated with signal transduction, cell migration, cell metabolism and cell proliferation. The analysis pointed some OA-specific cascades such as mTOR, Wnt and MAPK. Very importantly, we found that the exosomes derived from synovial fluid had property of endocytosis with chondrocytes leading to alterations of signalling process inside the chondrocytes. Hence, we spiculated that exosome derived from OA subjects may affect the metabolic process in the chondrocytes. The findings of our study confirmed that synovial fluid-isolated exosomes of OA subjects affected metabolic process such as inflammatory response and apoptosis in chondrocytes, and the findings were in agreement to reports published earlier which suggested that exosomes derived from IL-1β-stimulated SFBs could promote increased expression of ADAMTS and MMP-13 in chondrocytes compared to exosomes derived from normal subjects [39]. Our findings showed that SFB-derived exosomes overexpressing miR-214-3p suppressed inflammation in chondrocytes significantly compared to exosomes from control SFBs, indicating that these SFB-isolated exosomes can be important in regulating inflammatory response in the synovial space.

Literatures have confirmed that miRs are responsible for influencing the immune and inflammatory reactions targeting the associated genes responsible for apoptosis, differentiation and activation [40]. Hence, we spiculated that exosome-derived miRs collected from synovial fluid of OA subjects may inhibit joint remodelling and bone degeneration. As it was documented earlier that miR-214-3p is associated with OA and also our results found that miR-214-3p was significantly downregulated in SFBs of OA subjects, we studied the ability of exosomal miR-214-3p in influencing the pathogenesis of osteoarthritis. We hypothesized that miR-214-3p may inhibit the production of inflammatory cytokine after the chondrocytes were treated with sodium nitroprusside which is majorly used as a nitric oxide donor [41]. We found that overexpression of miR-214-3p resulted in suppression of sodium nitroprusside induced over expression of TNF-α, IL-1β and IL-6 and proliferation of chondrocytes also the SFB-miR-214-3p exosomes effectively suppressed the progression of OA. Further, we evaluated the effect of SFB-miR-214-3p exosomes on ACLT+ MMx-induced osteoarthritis rats. Prior to this, it was reported that the intra-articular exosomal delivery can lead to regeneration of cartilage and hence stop the progression of osteoarthritis [42, 43]. In the study, we evidenced that treatment of exosomes in the articular cartilage tissues suppressed the progression of OA significantly and also enhanced the cartilage regeneration thus preventing the cartilage damage in OA rats. Interestingly, SFB-miR-214-3p exosomes treatment significantly attenuated the loss of proteoglycan, irregular surface, inflammation of synovial joints and superficial fibrillation compared to rats receiving treatment of control exosomes which showed all these defects. The SFB-miR-214-3p exosomes-treated OA rats upon micro-CT analysis suggested high bone volume fraction values compared to rats treated with control exosomes, and the findings confirmed that treatment of SFB-miR-214-3p exosomes promoted healing in OA rats.

Though our findings indicated that exosomal miR-214-3p could significantly suppress the cartilage tissue degeneration, there are some limitations to our study such as more in-depth molecular analysis is needed for target gene confirmation, and also the patient sample size needs to be increased for more concrete results.

In conclusion, the findings of study confirmed that exosomes-derived miRs could be important regulators in progression of OA. SFB-miR-214-3p exosomes can target anti-inflammatory pathway thus suppressing the production of pro-inflammatory cytokines involved in progression of OA.

## Data Availability

All data generated or analysed during this study are included in this article. Further any enquiries can be directed to the corresponding author.
